# Preliminary Development of a Brief Cannabis Use Disorder Screening Tool: The Cannabis Use Disorder Identification Test Short-Form

**DOI:** 10.1089/can.2016.0022

**Published:** 2016-12-01

**Authors:** Marcel O. Bonn-Miller, Adrienne J. Heinz, Everett V. Smith, Raimondo Bruno, Simon Adamson

**Affiliations:** ^1^ Center for Innovation to Implementation, VA Palo Alto Health Care System, Menlo Park, California.; ^2^ National Center for PTSD, VA Palo Alto Health Care System, Menlo Park, California.; ^3^ Center of Excellence in Substance Abuse Treatment and Education, Philadelphia VA Medical Center, Philadelphia, Pennsylvania.; ^4^ Department of Psychiatry, University of Pennsylvania Perelman School of Medicine, Philadelphia, Pennsylvania.; ^5^ Department of Educational Psychology, University of Illinois at Chicago, Chicago, Illinois.; ^6^ School of Medicine (Psychology), University of Tasmania, Hobart, Australia.; ^7^ Department of Psychological Medicine, University of Otago, Christchurch, New Zealand.

**Keywords:** assessment, cannabis, cannabis use disorder, screening

## Abstract

**Introduction:** Rates of cannabis use disorder (CUD) among vulnerable populations have increased in recent years, highlighting a need to equip providers with an efficient screening tool.

**Materials and Methods:** A short form of the Cannabis Use Disorder Identification Test-Revised (CUDIT-R) was developed by using item response theory and traditional statistical methods, with data from two community samples of cannabis users representing two countries. Four item selection methods (Rasch regression, test characteristic curve, logistic regression, discriminant function analysis) were employed to identify the optimal three-item shortened version. The diagnostic ability of the short form was evaluated by using receiver operating characteristic curves.

**Results:** Using a cut score of 2, the 3-item CUDIT-Short Form (CUDIT-SF; reliability alpha=0.66, Sample 1; 0.80, Sample 2) identified 78.26% of participants in Sample 1 and 78.31% of participants in Sample 2 who met DSM-5 criteria for CUD, with 98% agreement in Sample 1 and 93% agreement in Sample 2 with the full CUDIT-R on CUD classifications using a cut score of 13. Specificity was 76.70 and 78.00 in Samples 1 and 2, respectively.

**Conclusions:** The CUDIT-SF may be useful in busy clinical settings for a stepwise screening. Further validation of this shortened version with larger samples and in different settings is warranted.

## Introduction

Cannabis is the most widely used illicit substance in the world.^1^ Indeed, though U.S. estimates of past year cannabis use problems (i.e., abuse, dependence) have remained relatively stable over the past 10 years, it is estimated that 7.6% of the U.S. population aged 18 or older have used cannabis in the past month, a significant increase over prior years.^2^ In Australia, estimates suggest that 35% of individuals aged 14 or older have used cannabis at some point in their life, with 5.3% and 3.5% of Australians reporting past-month and past-week use, respectively.^3^

With rates of cannabis use rising, particularly among certain vulnerable segments of the population (e.g., veterans^4^), there has been increased attention focused on the short- and long-term consequences associated with cannabis use. A recent review highlighted impairments in memory, motor coordination, and judgment as short-term consequences, and cognitive impairment and heightened risk of psychosis as long-term consequences of cannabis use.^5^ Beyond specific negative health effects, a considerable amount of research has now documented that heavy cannabis use, particularly when initiated during adolescence and used regularly, can lead to dependence and other associated negative consequences (e.g., poor life satisfaction or educational outcome.^5^ It is not, therefore, surprising that cannabis is second only to alcohol in terms of the primary substance for which individuals sought treatment in the past year.^2^

Unfortunately, although many treatments are available for individuals experiencing problems from cannabis use (e.g., contingency management^6^), a number of barriers preclude successful clinician diagnosis and treatment referral. In a study of Australian nurses and general practitioners, Norberg et al.^7^ highlighted significant barriers to cannabis screening and management, including lack of clinician time, interest, as well as knowledge and skills for treating cannabis problems.^7^ Consistent with these barriers, a study by Bonn-Miller et al.^4^ noted significant underdiagnosis of cannabis use disorders (CUDs) among a sample of U.S. military veterans seen within the Veterans Health Administration.^8^ More recent shifts in clinical practice models toward Screening Brief Intervention and Referral to Treatment (SBIRT) have been successful in addressing other health-risk behaviors, including problem drinking^9^; however one barrier to implementation for cannabis is the absence of a quick, reliable, and valid screening tool.

Equipping clinicians with a shortened screening tool can dramatically increase the feasibility of screening for substance use disorders in a more systematic fashion. For instance, the Alcohol Use Disorders Identification Test,^10^ a self-report measure developed by the World Health Organization to identify individuals with alcohol problems, was originally 10 items. Its length, therefore, rendered it less likely for providers to incorporate into routine patient interviews or include in general health history questionnaires.^11^ In response to this concern, the three-item AUDIT-C^11^ was developed and validated in the late 1990s and since 2006, it has been regularly administered to every patient seen in VA hospitals, the largest healthcare system in the United States.^12,13^ Similarly, provision of a valid, practical screening tool for CUD may help to combat perceived systemic and clinician-level barriers to diagnosing and treating problematic cannabis use.^14^ Specifically, implementation of a more formal and systematic assessment approach can serve to increase clinician confidence to detect CUD, make appropriate referrals, and engage clients in the treatment process—ultimately improving the overall system of care.

In an attempt to address some of the existing barriers to clinician screening, a team of Australasian (i.e., Australian and New Zealand) researchers created and later revised the Cannabis Use Disorders Identification Test (CUDIT^15,16^). The most recent version of the CUDIT (i.e., CUDIT-R^16^) is an 8-item screening tool that assesses individual cannabis consumption, problems associated with abuse, dependence, as well as psychological consequences (Appendix 1). The CUDIT-R shows high levels of specificity and sensitivity in terms of the identification of individuals with a current DSM-IV CUD.^17^ Adamson et al.^16^ were able to detect 91.3% of individuals with a CUD by using a cut point 13 or above on the CUDIT-R, whereas 90.0% of those without a CUD could be detected by using a cut point below 13.^16^

Although the sensitivity, specificity, and psychometric properties of the CUDIT-R are strong, the length of the CUDIT-R is not ideal for administration in busy clinical settings where providers face many competing priorities. Another limitation of the CUDIT-R is that it was developed and validated in accordance with DSM-IV.^17^ With the release of DSM-5,^18^ which contains significant changes to the classification of CUDs including the recognition and addition of cannabis withdrawal^19^ and craving, it is paramount that any new or revised screening tool is validated in accordance with the current diagnostic understanding of CUDs. A third limitation to existing work on the CUDIT-R relates to the population among which it has been validated. Though the CUDIT and CUDIT-R have been widely used in studies outside of Australasia (e.g., Ref.^20^), aside from the validation of a modified 14-item version of the CUDIT among a Swiss sample,^21^ analyses of sensitivity and specificity have only been conducted among Australasian populations. Accordingly, the present study aimed at filling existing gaps in the literature by utilizing item response theory (IRT) to identify and test a shortened version of the CUDIT-R, which could be used in busy clinical settings (e.g., primary care) to quickly and accurately identify individuals meeting DSM-5^18^ criteria for CUD, among two populations: medical cannabis users in the United States, and a community sample of cannabis users in Australia.

Approaches to measurement employing IRT, and Rasch models in particular, demonstrate several advantages over methods based on classical test theory (e.g., Ref.^22^). One such advantage, relevant to the current study, is that Rasch models yield a standard error for every parameter estimated (i.e., item and person-level parameters). This stands in contrast to classical test theory, whereby researchers usually rely on a single standard error of measurement applied equally to all ability levels (e.g., levels of problematic cannabis use^22^). Such an approach does not allow for examination of the expected degree of measurement error on an item-by-item basis at different ability levels (i.e., levels of problematic cannabis use). Another advantage is the ease with which Rasch and IRT models are often utilized to facilitate a smaller selection of items to assess a latent trait (e.g., proficiency), thereby reducing test length and increasing efficiency (e.g., Refs.^23–25^). Accordingly, the objective of the current study was to employ IRT, with an emphasis of Rasch models, to select a subset of CUDIT-R items that optimized the short form's capacity to reliably detect problematic cannabis use (i.e., the latent trait).

## Materials and Methods

### Participants

Participants were two community samples of cannabis users. Sample 1 was recruited from a medical cannabis facility in the United States, and Sample 2 was recruited from a community sample of regular (past year) recreational cannabis consumers in Australia. Sample 1 consisted of 207 participants (24% female; M_age_=41.17 years, SD_age_=14.79; 68% Caucasian, 8% Hispanic, 7% African-American/non-Hispanic, 3% African-American/Hispanic, 3% Asian-American, 11% other). Sample 2 consisted of 369 participants (37% female; M_age_=28.13 years, SD_age_=10.91); data on ethnicity were not available for this sample.

### Measures

Cannabis use quantity was assessed with a single item in each sample. Participants in Sample 1 were asked, “How much cannabis (grams) do you usually use per week?” (1=1 g, 2=2 g, 3=3–5 g, 4=6–8 g, 5=9–12 g, 6=more than 12 g), whereas participants in Sample 2 were asked, “On a typical day when you use cannabis, on average, how many cones, bongs, or joints do you normally have?”

The Cannabis Use Disorder Identification Test-Revised (CUDIT-R^16^) is an eight-item self-report questionnaire that assesses problematic cannabis use within the past 6 months (Appendix 1). Items assess consumption frequency, time spent stoned, cannabis abuse (e.g., use in hazardous situations) and cannabis dependence (e.g., not able to stop using, spending a lot of time obtaining, using, or recovering from use), negative consequences of use (e.g., problems with memory and concentration), and intention to cut down or stop use. Scores can range from 1 to 32, with a cut-off score of 13 indicative of a DSM-IV diagnosis of CUD (dependence).^16^ Criteria for DSM-IV cannabis abuse are met if one or more of four symptoms are endorsed, and for dependence if three or more of seven symptoms are endorsed.

For Sample 1, past-6 month DSM-5 CUD was assessed via a self-report questionnaire derived from the Structured Clinical Interview, Non-Patient Version for DSM-IV (SCID-I-N/P^26^). Consistent with changes for DSM-5 criteria, a positive diagnosis of CUD could have also included withdrawal.^19^ Though DSM-5 CUD scoring rules were utilized, assessment of CUD in this study did not include craving due to the timing of the study, thus we refer to these modified DSM-5 criteria (i.e., without craving) from here forward as DSM-5-M.

For Sample 2, the presence of past 6 month CUD was established by using an amended version of the Alcohol Use Disorder and Associated Disabilities Interview Schedule (AUDADIS-IV^27^) supplemented with questions from the Comprehensive International Diagnostic Interview (CIDI^28^) to assess both the presence of craving and, independently, the presence of each of the seven signs and symptoms of cannabis withdrawal relevant to DSM-5, using the withdrawal criteria as specified in the DSM-5 (including withdrawal relief). Consistent with DSM-5 scoring rules, participants in both samples met criteria for CUD if they endorsed two or more symptoms (10 symptoms assessed in Sample 1; 11 symptoms in Sample 2).

### Procedure

In Sample 1, adults receiving cannabis at a medical dispensary were approached to participate in research. Participants older than the age of 18 who provided written informed consent to participate in the study completed the SCID-I-N/P assessing for CUD and the CUDIT-R. All procedures were approved by the Stanford and VA Palo Alto Institutional Review Boards. In Sample 2, individuals residing in Australia and who were active cannabis smokers in the past 12 months were invited to complete an internet-based survey, comprising the modified AUDADIS-IV and CIDI items assessing DSM-5 CUD as well as the CUDIT-R. All study procedures were approved by the Tasmania Social Sciences Human Research Ethics Committee.

### Data analyses

Descriptive statistics and internal consistency (Cronbach alpha) for the full-scale CUDIT-R were calculated for both samples. In Sample 1, 12 participants had missing data and a full CUDIT-R score could not be calculated. In Sample 2, 3 participants responded 0 to item 1 (i.e., reported no use in past 6 months). These participants were included in the descriptive statistics but removed from Kappa, percent agreement, and receiver operating curve (ROC) analyses (*n*=195, Sample 1; *n*=366, Sample 2).

Item thresholds and person parameters were calculated with the Rasch partial credit model^29^ using WINSTEPS version 3.23.^30,31^ The partial credit model was applied to allow for different scoring structures and model parameters for each item (items 1 and 8 have different rating scales, see results). Requests for detailed results of evaluating Rasch model assumptions and the evaluation of rating scale functioning can be made directly to the corresponding author.

Classification consistency (Cohen's Kappa^32^) and percent agreement between the CUDIT-R full form recommended cut score of 13 and DSM-5/5-M diagnosis of CUD were examined first. Of note, the cut score of 13 is based on DSM-IV for cannabis dependence. Next, area under the ROC and estimates of sensitivity and specificity derived using Medcalc^33^ were used to determine optimal cut points. Sensitivity was the proportion of individuals correctly classified as positive for CUD based on DSM-5/5-M criteria. Specificity was the proportion of individuals correctly classified as negative for CUD based on DSM-5/5-M criteria.

#### Methods of item selection

Four different methods of item selection were employed to identify the most optimized short form of the CUDIT-R.

(1) Rasch regression.^34^ In this method, the people are first anchored (fixed) at their dependent variable values (DSM-5/5-M CUD positive classification=1; negative classification=0). Next, WINSTEPS finds the best combination of independent variables (the eight CUDIT-R items) for predicting the anchored dependent variable values. The best three-item combination was determined by using item fit statistics and principal components analysis of standardized residuals (requests for detailed results of evaluating Rasch model assumptions using item fit statistics and principal components analysis of standardized residuals can be made directly to the corresponding author). The purported advantage of Rasch regression over traditional regression methods is the lack of assumptions regarding the distribution properties of the dependent and independent variables assumed by traditional regression methods.

(2) Test characteristic curve (TCC) method.^23^ The statistical item information from a Rasch calibration of all eight items was combined to form a TCC. The TCC provides the amount of statistical information (precision) available at all ability levels (e.g., cannabis use problems). The TCC yielded maximum information at 0.775 logits. The ROC with the full form in Sample 1 demonstrated good sensitivity (80.4) and specificity (74.8) for DSM-5-M CUD at a cut score of 10. Thus, the goal was to mirror the overall TCC with the short form. To do this, item information functions (IFF) from each item were examined and the three items with the IFF that most closely matched the full CUDIT-R TCC (i.e., had the maximum information at 0.775 logits) were selected.

(3 and 4) Logistic regression and discriminant function analysis (e.g., Ref.^35^). Three logistic regressions were performed in SPSS by using different item selection methods: (1) Forward Stepwise using the Wald statistic, (2) Forward Stepwise using the Likelihood Ratio, and (3) Forward Stepwise using conditional item selection. For the Discriminant Function Analysis, the stepwise method in SPSS was utilized. For all four analyses, the DSM-5/5-M CUD was entered as the dependent variable, and the independent variables were all eight items of the CUDIT-R. The first three items selected were retained for the short form. We have grouped all four of these analyses here as the same three items that were selected across all analyses.

Area under the ROC and estimates of sensitivity and specificity for DSM-5-M/DSM-5 CUD were calculated for each item selection method to identify the most optimized short form. The same procedures were then performed with Sample 2 to determine whether results could be acceptably replicated. After items were selected for the CUDIT-short form, Kappa and percent agreement between the CUDIT-short form recommended cut score and DSM-5/5-M diagnosis of CUD was examined in each sample. Construct validity for the CUDIT-short form was assessed in two ways. First, correlations were performed to examine the relation between scores from the full CUDIT-R and the new short form. Second, agreement between the short and full forms' classifications (CUD or no CUD) was examined by using percent agreements and Kappa indices. Third, correlations were conducted to assess the relation between CUDIT-SF score and DSM-5-M/DSM-5 symptom count. Fourth, in Sample 2 only (Sample 1 contained modified DSM-5 symptom criteria), an ANOVA was conducted to determine the extent to which CUDIT-SF score differed as a function of diagnostic severity for DSM-5 CUD (no diagnosis, mild, moderate, severe).

## Results

### Descriptive statistics

In Sample 1, participants reported using an average of 3–5 g of cannabis per week (30% reported 3–5 g; M=3.17, SD=1.43; *n*=200). In Sample 2, participants used an average of 5.89 (SD=9.38; *N*=358) cones, bongs, or joints on a typical day when they used cannabis. In Samples 1 and 2, 47% (*n*=92) and 46% (*n*=168) of participants met DSM-5-M/DSM-5 criteria for CUD, respectively. In Sample 1, the average number of DSM-5-M symptoms endorsed was 1.84 (SD=1.79; 10 possible symptoms) and in Sample 2, the average number of DSM-5 symptoms was 2.27 (SD=2.74; 11 possible symptoms). Mean scores on the CUDIT-R were 10.65 (SD=5.09; *n*=195) and 10.71 (SD=7.05; *n*=369) for Samples 1 and 2, respectively. Using the recommended cut score of 13 or higher on the full-form CUDIT-R, which is a cut score based on DSM-IV dependence, 27% of Sample 1 (*n*=53) and 33% of Sample 2 (*n*=122) were identified as at-risk for DSM-5 CUD. Internal consistency for the full version of the CUDIT-R was 0.71 in Sample 1 and 0.82 in Sample 2. Table 1 provides descriptive statistics on the number of participants from each sample who endorsed each item (i.e., score of one or higher) on the CUDIT-R.

**Table 1.** **Endorsement of Individual CUDIT-R Items Across Samples**

	Sample 1, *n*=207 (%)	Sample 2, *n*=369 (%)
1. How often do you use cannabis?	100	99
2. How many hours were you “stoned” on a typical day when you had been using cannabis?	96	92
3. How often during the past 6 months did you find that you were not able to stop using cannabis once you had started?	24	28
4. How often during the past 6 months did you fail to do what was usually expected from you because of using cannabis?	27	37
5. How often in the past 6 months have you devoted a great deal of your time to getting, using, or recovering from cannabis?	35	42
6. How often in the past 6 months have you had a problem with your memory or concentration after using cannabis?	60	60
7. How often do you use cannabis in situations that could be physically hazardous, such as driving, operating machinery, or caring for children?	27	34
8. Have you ever thought about cutting down, or stopping, your use of cannabis?	54	64

Percentage reflects number of individuals who endorsed the presence of any item (i.e., did not endorse zero).

CUDIT-R, Cannabis Use Disorder Identification Test-Revised.

### Kappa and percent agreement for full-form CUDIT-R

A chi-square test revealed that individuals in Sample 1 who met DSM-5-M criteria for CUD were significantly more likely to have a full-scale CUDIT-R score of 13 or higher χ^2^ (2, *n*=195)=64.96, *p*<0.001. In Sample 1, kappa was low (0.53) and among the 92 participants who met DSM-5-M criteria for CUD, only 54% (*n*=50) scored a 13 or higher on the CUDIT-R. Of the 103 participants who did not meet DSM-5-M criteria for CUD, 97% (*n*=100) had a full-scale CUDIT-R score of less than 13. In Sample 2, a chi-square test revealed that individuals who met DSM-5 criteria for CUD were significantly more likely to have a full-scale CUDIT-R score of 13 or higher χ^2^ (2, *n*=366)=103.45, *p*<0.001. In Sample 2, kappa was also low (0.52) and among the 166 participants who met DSM-5 criteria for CUD, only 61% (*n*=101) scored a 13 or higher on the CUDIT-R. Of the 200 participants who did not meet DSM-5 criteria for CUD, 90% (*n*=179) had a full-scale CUDIT-R score of less than 13. A raw cut score of 13 was better at detecting true negatives in Sample 1 than in Sample 2 (Sample 1–97%, Sample 2–90%). However, this cut score of 13 was better at detecting true positives in Sample 2 than in Sample 1 (Sample 1–54%, Sample 2–61%). Given that DSM-5 thresholds for the presence of a CUD diagnosis are much lower than those for DSM-IV, results were in line with expectations that many of the individuals who met DSM-5 CUD would have a lower CUDIT-R score than that required for the cut-off score for DSM-IV dependence (CUDIT-R=13).

### CUDIT-R full-form ROC

In Sample 1, a cut-off of 10 yielded the highest combined values of a sensitivity of 80.43 and a specificity of 74.76 (AUC=0.88) for DSM-5-M CUD. In contrast, a cut-off of 13 (based on DSM-IV Cannabis Dependence) on the full version of the CUDIT-R yielded a sensitivity of 54.35 and a specificity of 97.09 for DSM-5-M CUD. In Sample 2, a cut-off of 9 yielded the highest combined values of a sensitivity of 80.72 and a specificity of 72.00 (AUC=0.84) for DSM-5 CUD. A cut-off of 13 yielded a sensitivity of 60.84 and a specificity of 89.50 for DSM-5 CUD in Sample 2.

### Item selection

Results from the Rasch regression revealed that items 4, 5, and 6 were the best fitting items that also differentiated between participants with different levels of cannabis use problems in Samples 1 and 2. Results from the TCC method indicated that items 3, 5, and 6 combined to yield a TCC that was the most similar to the TCC of the full eight-item CUDIT-R. Results from the three LR and the DFA indicated that items 3, 6, and 8 (Sample 1) and items 5, 6, and 8 (Sample 2) were able to best discriminate individuals with and without CUD. Requests for detailed results of these analyses can be made directly to the corresponding author.

### ROC with CUDIT-short form

Using the items identified with the Rasch regression (4, 5, and 6), ROC analyses with an optimal cut point of two indicated that sensitivity was 73.91 and specificity was 72.82 in Sample 1 (AUC=0.81) for DSM-5-M CUD; sensitivity was 80.12 and specificity was 72.00 in Sample 2 (AUC=0.84) for DSM-5 CUD. ROC analyses using items identified with the TCC method with an optimal cut point of two indicated that sensitivity was 78.26 and specificity was 76.70 for DSM-5-M CUD in Sample 1 (AUC=0.84) and in Sample 2, sensitivity was 78.31 and specificity was 78.00 (AUC=0.85) for DSM-5 CUD. Finally, using items identified with the LR/DFA (items 3, 6, and 8: Sample 1; items 5, 6, and 8: Sample 2), ROC analyses with an optimal cut point of two indicated that sensitivity was 78.26 and specificity was 70.87 for DSM-5-M CUD in Sample 1 (AUC=0.84); with an optimal cut point of three, sensitivity was 81.33 and specificity was 70.00 for DSM-5 CUD in Sample 2 (AUC=0.85). Please see Table 2 for details and confidence intervals.

**Table 2.** **Performance of Optimal CUDIT Cut Points with Different Item Selection Methods in Identifying DSM-5-M/5 Cannabis Use Disorder**

	Sensitivity	Specificity	AUC	CI
CUDIT full form—standard cut 13				
Sample 1	54.35	97.09	0.88	0.83–0.92
Sample 2	60.84	89.50	0.84	0.80–0.88
CUDIT full form—alternative cut score				
Sample 1 (cut 10)	80.43	74.76	0.88	0.83–0.92
Sample 2 (cut 9)	80.72	72.00	0.84	0.80–0.88
Rasch regression				
Sample 1 (cut 2; items 4, 5, 6)	73.91	72.82	0.81	0.75–0.87
Sample 2 (cut 2; items 4, 5, 6)	80.12	72.00	0.84	0.80–0.88
Test characteristic curve				
Sample 1 (cut 2; items 3, 5, 6)	78.26	76.70	0.84	0.78–0.89
Sample 2 (cut 2; items 3, 5, 6)	78.31	78.00	0.85	0.81–0.88
Logistic regression/discriminant function analysis				
Sample 1 (cut 2; items 3, 6, 8)	78.26	70.87	0.84	0.78–0.89
Sample 2 (cut 3; items 5, 6, 8)	81.33	70.00	0.85	0.81–0.89

^a^ Sample 1, *n*=195; Sample 2, *n*=366. DSM-5/5-M CUD is defined as two or more criteria.

AUC, area under the curve; CI, confidence interval; CUD, cannabis use disorder.

In Samples 1 and 2, the TCC item-selection method appeared to provide the highest combined sensitivity and specificity. The TCC method also had lower sensitivity but higher specificity than the full-scale CUDIT-R with the alternative cut score for both samples. The items selected with the Rasch regression and LR/DFA demonstrated similar or lower sensitivity and lower specificity when compared with items identified with TCC. Ultimately, the TCC method provided the best balance between sensitivity and specificity and items 3, 5, and 6 were selected to comprise the final CUDIT-SF. A cut score of two was deemed appropriate. Please see Appendix 2 for a copy of the CUDIT-SF with scoring instructions. Internal consistency for the CUDIT-SF was 0.66 in Sample 1 and 0.80 in Sample 2.

### Correlation and percent agreement of CUDIT-R full form to CUDIT-short form; relations between CUDIT-SF and DSM-5 symptom count and severity category

Spearman correlations indicated that the CUDIT-SF was correlated at 0.84 with the CUDIT-R full form in both Samples 1 and 2. In Sample 1, 49% of participants had a score of two or higher on the CUDIT-SF and in Sample 2, 48% had a score of two or higher. In Sample 1, kappa was 0.55 and among the 92 participants who met DSM-5-M criteria for CUD, 78% (*n*=72) scored a two or higher on the CUDIT-SF. A chi-square test revealed that individuals in Sample 1 who met DSM-5-M criteria for CUD were significantly more likely to have a full-scale CUDIT-SF score of two or higher χ^2^ (2, *n*=195)=58.73, *p*<0.001. In Sample 2, kappa was 0.56 and among the 166 participants who met DSM-5 criteria for CUD, 78% (*n*=130) scored a two or higher on the CUDIT-SF. A chi-square test revealed that individuals in Sample 2 who met DSM-5 criteria for CUD were significantly more likely to have a full-scale CUDIT-SF score of two or higher χ^2^ (2, *n*=366)=115.34, *p*<0.001. In Sample 1, kappa was 0.54 and among the 53 participants who scored 13 or higher on the CUDIT-R full form (a cut point based on DSM-IV Cannabis Dependence established in a previous, smaller sample—Ref.^16^), 52 (98%) scored a two or higher on the CUDIT-SF. In Sample 2, kappa was 0.62 and of the 122 participants who scored 13 or higher on the CUDIT-R full form, 114 (93%) scored a two or higher on the CUDIT-SF.

Spearman correlations indicated that CUDIT-SF score and DSM-5-M/DSM-5 symptom count were strongly and positively associated in Sample 1 (ρ=0.67, *p*<0.001) and Sample 2 (ρ=0.72, *p*<0.001). In Sample 2, an ANOVA indicated that CUDIT-SF scores significantly differed for each DSM-5 CUD diagnostic severity category (F [3, 368]=174.01. *p*<0.001 [η^2^=0.59]; no diagnosis=1.03, [SD=1.60], mild=2.71 [SD=2.07], moderate=5.06 [SD=3.35], severe=8.52 [SD=3.07]).

## Discussion

The present investigation sought to identify and test a shortened version of the eight-item CUDIT-R in samples from two different countries (i.e., the United States and Australia) by using IRT and traditional statistical methods. Analyses revealed a three-item measure (CUDIT-SF), comprising items 3, 5, and 6 from the CUDIT-R (Appendix 2), which demonstrated (with a cut point of two) a sensitivity of 78.26 and 78.31 and a specificity of 76.70 and 78.00 in the prediction of DSM-5-M/5 CUD in U.S. and Australian samples, respectively. The resulting CUDIT-SF is a tool that provides a solution for significant barriers to cannabis screening (e.g., time to administer, clinician knowledge/training),^7^ and it can quickly be administered in busy clinical settings to identify, with adequate accuracy, individuals who likely meet criteria for DSM-5 CUD.

The items that emerged for the CUDIT-SF focused less on the frequency of use or time spent intoxicated, but rather the inability to stop using, consequences resulting from use (i.e., cognitive effects), as well as the time typically spent acquiring, using, or recovering from the effects of cannabis. Though this may not be surprising given that problems associated with use is a defining factor for a diagnosis of CUD, it is somewhat surprising that a question that, at least partially, inquires about the time spent obtaining cannabis (item 5) would be associated with problematic use among a sample who obtains cannabis from a medical dispensary (i.e., Sample 1). Although the three selected CUDIT-SF items appear to provide the best combination of specificity and sensitivity, all item selection methods yielded similar results. As such, clinicians in certain settings may find the contents of an alternative item set more useful with their specific clinical population. Further examination of the sensitivity and specificity of the CUDIT-SF (potentially with other item combinations) among other cannabis populations, such as individuals in primary care or people obtaining cannabis in states where recreational and medical use has been legalized (i.e., Colorado and Washington), would be informative.

Another caveat to the present study was the observation that the original eight-item CUDIT-R scale performed relatively poorly with a cut point of 13 in the prediction of DSM-5 defined CUD. The poor observed Kappa, sensitivity, and specificity are not entirely surprising given that the CUDIT-R cut point of 13 was determined according to DSM-IV criteria for cannabis abuse/dependence among a clinical sample.^16^ Specifically, DSM-IV cannabis dependence requires a higher threshold of symptom endorsement relative to DSM-5 CUD. Indeed, the present study suggests that utilizing a cut point of 10 for the CUDIT-R may be more appropriate for the identification of individuals from non-clinical samples who are likely to have DSM-5 CUD. Given that cut points were based on “mild” DSM-5 CUD (i.e., endorsement of two or more symptoms), future research should determine appropriate cut points for the CUDIT-R based on moderate levels of CUD (i.e., endorsement of 4–5 symptoms).

Though not central to the present investigation, other caveats to consider in future modifications of the CUDIT-R include whether to (1) retain the option of “never” in question 1 given the cannabis use-screening question at the top of the measure, (2) modify the rating scales so as to clarify and/or streamline response options (e.g., provide 3-point instead of 5-point scales by combining the 0 and 1 and 3 and 4 categories), or (3) revise the numbers corresponding to response items in question 8, from (0, 2, 4) to (0, 1, 2). Future work could also benefit from modifying the eight-item CUDIT-R based on the present findings, as well as replicating the ROC analyses among other cannabis-using samples (e.g., adolescents).

Though the present study contributes a shortened screening measure for the identification of individuals with CUD, it is not without limitations. First, rates of CUD observed in the two samples (47% and 46%, respectively) were significantly higher than those reported for lifetime prevalence in large epidemiological studies (e.g., Ref.^36^). However, observed rates were generally consistent with rates of CUDs observed among regular users (as were both samples in the current study^37^), particularly given that DSM-5 thresholds for the presence of a CUD diagnosis are much lower than for DSM-IV. Future studies with different samples should examine how psychometric characteristics of CUDIT-R items compare with this and other IRT analyses of cannabis involvement (e.g., Ref.^38^). Second, although a strength of the study was the validation of the CUDIT-SF among differing populations from two countries, ethnicity data were not collected in Sample 2 (which is common in Australian drug research) and the assessment of DSM-5 CUD in Sample 1 did not include craving, which is necessary for the accurate diagnosis of DSM-5 CUD. As such, we cannot be sure as to how representative these samples are of the general community with regard to other co-occurring substance use or demographic characteristics such as education and income. The lack of assessment of craving in Sample 1 is of particular concern for analyses that involve frequency counts of diagnostic criteria. Future work would benefit from employing consistent, diagnostically accurate, and expanded assessments across samples. However, support for the generalizability of the final model is bolstered by the appropriate range of scores observed in the data and the consistency of the results across the two independent samples.

Third, data in Sample 1 were collected in-person via paper-and-pencil, whereas data in Sample 2 were collected remotely via an internet-based survey. Although it is unclear how differential data collection methods may have impacted survey responding, future work would benefit from method consistency across samples. Finally, the present study was limited by its cross-sectional design. Although the CUDIT-R has been found to reliably detect clinically significant changes in CUD symptoms throughout the course of treatment,^39^ a prospective study is required to examine the potential utility of the CUDIT-SF in the identification of those at risk for developing cannabis use problems over time.

## Abbreviations Used

AUDADIS-IV: Alcohol Use Disorder and Associated Disabilities Interview Schedule
CIDI: Comprehensive International Diagnostic Interview
CUD: Cannabis Use Disorder
CUDIT-R: Cannabis Use Disorder Identification Test-Revised
CUDIT-SF: Cannabis Use Disorder Identification Test-Short Form
IFF: item information functions
IRT: item response theory
ROC: receiver operating curve
SBIRT: Screening Brief Intervention and Referral to Treatment
SCID-I-N/P: Structured Clinical Interview, Non-Patient Version for DSM-IV
TCC: test characteristic curve

## Appendix 1: CUDIT-R

Have you used any cannabis over the past 6 months? YES/NO

If YES, please answer the following questions about your cannabis use. Circle the response that is most correct for you in relation to your cannabis use over the past 6 months:

**Table:**

1.	How often do you use cannabis?				
	Never	Monthly or less	2–4 times a month	2–3 times a week	4 or more times a week
	0	1	2	3	4
2.	How many hours were you “stoned” on a typical day when you had been using cannabis?				
	Less than 1	1 or 2	3 or 4	5 or 6	7 or more
	0	1	2	3	4
3.	How often during the past 6 months did you find that you were not able to stop using cannabis once you had started?				
	Never	Less than monthly	Monthly	Weekly	Dally or almost daily
	0	1	2	3	4
4.	How often during the past 6 months did you fail to do what was usually expected from you because of using cannabis?				
	Never	Less than monthly	Monthly	Weekly	Daily or almost daily
	0	1	2	3	4
5.	How often in the past 6 months have you devoted a great deal of your time to getting, using, or recovering from cannabis?				
	Never	Less than monthly	Monthly	Weekly	Daily or almost daily
	0	1	2	3	4
6.	How often in the past 6 months have you had a problem with your memory or concentration after using cannabis?				
	Never	Less than monthly	Monthly	Weekly	Daily or almost daily
	0	1	2	3	4
7.	How often do you use cannabis in situations that could be physically hazardous, such as driving, operating machinery, or caring for children?				
	Never	Less than monthly	Monthly	Weekly	Dally or almost daily
	0	1	2	3	4
8.	Have you ever thought about cutting down, or stopping, your use of cannabis?				
	Never		Yes, but not in the past 6 months		Yes, during the past 6 months
	0		2		4

## Appendix 2: CUDIT-SF

Do you currently use cannabis? YES/NO

IF YES:

**Table:**

1. How often during the past 6 months did you find that you were not able to stop using cannabis once you had started?				
Never	Less than monthly	Monthly	Weekly	Daily or almost daily
0	1	2	3	4
2. How often in the past 6 months have you devoted a great deal of your time to getting, using, or recovering from cannabis?				
Never	Less than monthly	Monthly	Weekly	Daily or almost daily
0	1	2	3	4
3. How often in the past 6 months have you had a problem with your memory or concentration after using cannabis?				
Never	Less than monthly	Monthly	Weekly	Daily or almost daily
0	1	2	3	4

Total Score _______

Positive Screen=2 or higher
